# Understanding Emergency Department Experiences of People Experiencing Homelessness to Inform Equitable Medical Education

**DOI:** 10.51894/001c.164402

**Published:** 2026-08-01

**Authors:** Naiyah Thompson, Shambhvi Ojha, Dyelle Frederick, Marissa Goudeseune, Dana Midani, Carolina B. Restini, Richard Bryce

**Affiliations:** 1 College of Osteopathic Medicine, Michigan State University

## 48

### Background

People experiencing homelessness (PEH) face significant barriers in healthcare, relying on emergency departments (EDs) up to five times more than the general population. They experience higher rates of chronic diseases, mental health issues, and traumatic injuries, compounded by difficult social circumstances. It is important to consider interventions that train healthcare workers in patient-centered care and address these disparities through street medicine initiatives. Understanding PEH’s experiences is critical to improving trust and equity in care, aligning with osteopathic principles of viewing patients within their social contexts.

### Objective

This cross-sectional qualitative study, conducted through a street medicine initiative, explores perceptions of bias faced among PEH in emergency room settings. Objectives include: 1) identifying perceived bias; 2) examining medical mistrust and its impact on trust-building in medical practice; and 3) identifying factors contributing to these experiences to inform equitable medical education strategies.

### Methods

This study (IRB-00011655) used semi-structured interviews (10–15 minutes) and an adapted 12-item validated Group-Based Medical Mistrust Scale (MGBMMS), during Detroit Street Care outreach. Housed (control, N=5) and unhoused (PEH, N=7) adults (> 18 years) who visited the ED within the past year and were capable of providing informed consent were included. Interviews assessed bias and trust. The MGBMMS used the Mann-Whitney test to compare mistrust between groups.

### Results

Housed participants reported 46% negative and 26% positive ED experiences, while PEH reported 29% negative and 47% positive experiences. 42% of unhoused participant saw a social worker. Negative experiences for PEH surrounded feeling overlooked, housed individuals felt the hospital was too money oriented. Transportation remained a common barrier (41%) despite assistance. MGBMMS results showed no significant difference in medical mistrust between groups.

### Conclusions

PEH reported more positive ED experiences, attributed to compassionate staff interactions. However, PEH noted communication gaps and low engagement with social workers, highlighting areas for improvement. The current sample size does not allow for definitive conclusions, but data suggests progress in Detroit’s healthcare system for vulnerable populations. Though more research is needed, it is clear that medical education must address the importance of integrating medical and community resources to improve patient trust, promote equity, and advance patient-centered care.

